# Antioxidants as Protection against Reactive Oxygen Stress Induced by Formaldehyde (FA) Exposure: A Systematic Review

**DOI:** 10.3390/biomedicines12081820

**Published:** 2024-08-10

**Authors:** Loredana Beatrice Ungureanu, Cristina Mihaela Ghiciuc, Cornelia Amalinei, Carmen Ungureanu, Cristina Gabriela Petrovici, Raluca Ștefania Stănescu

**Affiliations:** 1 Morphopathology, Department of Morpho-Functional Sciences I, Faculty of Medicine, Grigore T. Popa University of Medicine and Pharmacy, 16 Universitatii Street, 700115 Iasi, Romania; loredana.ungureanu@umfiasi.ro (L.B.U.); carmen.ungureanu@umfiasi.ro (C.U.); 2 Pharmacology, Clinical Pharmacology and Algeziology, Department of Morpho-Functional Sciences II, Faculty of Medicine, Grigore T. Popa University of Medicine and Pharmacy, 16 Universitatii Street, 700115 Iasi, Romania; 3 Histology, Department of Morpho-Functional Sciences I, Faculty of Medicine, Grigore T. Popa University of Medicine and Pharmacy, 16 Universitatii Street, 700115 Iasi, Romania; 4 Infectious Disease, Department of Medical II, Faculty of Medicine, Grigore T. Popa University of Medicine and Pharmacy, 16 Universitatii Street, 700115 Iasi, Romania; cristina.petrovici@umfiasi.ro; 5 Biochemistry, Department of Morpho-Functional Sciences II, Faculty of Medicine, Grigore T. Popa University of Medicine and Pharmacy, 16 Universitatii Street, 700115 Iasi, Romania; raluca.stanescu@umfiasi.ro

**Keywords:** formaldehyde, oxidative stress, antioxidants, histopathology

## Abstract

Background and Objectives: Formaldehyde induces oxidative stress and is carcinogenic, particularly squamous cell carcinoma of the nasopharyngeal area. Around us, in exhaust gases, cigarette smoke, and various industrial products, FA primarily affects the respiratory tract and other organs like the cornea, liver, kidneys, brain, and cardiovascular system. This study aims to determine if antioxidants can mitigate FA’s harmful effects. Materials and Methods: Several databases, including PubMed, Science Direct, Springer, and Wiley, were systematically searched. Research publications on antioxidants mitigating FA-induced oxidative damage were included, but reviews and articles lacking complete texts were excluded. SYRCLE’s risk of bias tool for animal studies has been used. Tables were used for data synthesis. Out of 8790 articles, 35 publications detailing tissue homogenate for biochemical analysis, standard hematoxylin–eosin staining, and immunohistochemistry markers for histopathological and immunohistochemical diagnosis were selected. Most studies were case–control studies, utilizing rat or mouse models. Additionally, one cohort study on industrial workers was analyzed. Conclusions: Antioxidants, including plant extracts, vitamins, and pigments, can prevent or heal FA-induced lesions. However, human studies, particularly biopsies, remain challenging, and animal trials are limited. Further research is needed to confirm FA’s long-term effects and optimize antioxidant dosages.

## 1. Introduction

Formaldehyde (FA), a carcinogen, induces cell damage and oxidative stress via reactive oxygen species (ROS) [1,2,3,4]. FA is classified by the International Agency for Research on Cancer (IARC) as a class A carcinogen [3,5,6,7,8,9]. FA has been implicated as a human carcinogen, particularly in nasopharyngeal carcinoma and leukemia [10,11,12]. The carcinogenic effect has been demonstrated in rats and, to a lesser extent, in mice [13]. The effect, which occurred only after long-term exposure (two years), was particularly represented by nasopharyngeal squamous cell carcinoma [13]. Several studies and meta-analyses have investigated the carcinogenic effects of FA in humans [14,15,16,17,18,19,20]. The association between FA exposure and myeloid leukemia was established by the IARC, based on the epidemiological finding of an increased number of workers who developed leukemia [6,14], but the leukemogenic effect was only observed in human cell cultures, not in vivo [14]. For ethical reasons, there is a lack of studies on the carcinogenic effect of FA in humans. The association between FA and lung cancer is inconclusive due to concurrent exposure to other substances and similar risk levels in control groups [16]. The risk of non-Hodgkin’s lymphoma is also reduced [17]. A 2019 review of a small number of studies found no causal association between FA exposure and myeloid leukemia [18], but a study on Korean workers showed a significant dose-dependent relationship for several cancers (nasopharyngeal carcinoma, lymphohematopoietic malignancies, and non-Hodgkin’s lymphoma) [19]. An increased risk of nasopharyngeal carcinoma was observed in a long-term exposure of 34 years [20]. Overall, a clear conclusion on FA carcinogenic effects has not been reached.

FA is the simplest of the aldehydes [10,21], is water soluble [22], and can become gaseous at room temperature [22]. It is a toxic substance produced endogenously [10,12] and metabolized in mitochondria [11] or from exogenous sources [10]. Approximately 90% of FA in the body is endogenous and 10% is exogenous [23].

FA is either exhaled as carbon dioxide or metabolized to formic acid [11,24] in the liver [12,22] and erythrocytes [22] and excreted in the feces or urine [22]. ROS and FA are involved in a vicious circle by stimulating each other’s synthesis (ROS produce oxidative stress, leading to FA synthesis, while FA is an inducer of oxidative stress with increased ROS production) [24]. Individual responses to FA exposure vary widely, with some individuals remaining asymptomatic and others developing organ damage even at low doses [22]. Inhalation of FA is highly irritating to the nasal and ocular mucosa and less irritating to the lower respiratory tract [25]. Prolonged exposure to FA can damage various organs and systems in the body, and FA exposure can harm the liver, kidneys, and cerebral cortex [7] and disrupt lipid metabolism [26]. FA also triggers allergic reactions, such as allergic rhinitis, chemical sensitivities, or bronchial asthma [27]. FA also triggers cardiovascular effects such as the induction of atherosclerosis and myocardial infarction [11,24] or neurological effects such as the potential to cause degenerative diseases, such as dementia, and, in particular, Alzheimer’s disease [11,21,28], multiple sclerosis [21], and Parkinson’s disease [29], as well as manifestations such as headaches, sleep disturbances, memory impairment [30,31], dizziness [5,30], severe fatigue, thirst, irritability, lethargy, behavioral and sensory/emotional disturbances [30], and cognitive impairment [28]. In the reproductive system, FA can cause primary and secondary infertility [32] by reducing spermatogenesis or affecting menstrual function [30], as well as pregnancy complications, such as spontaneous abortion [11] or anemia, malformations, and low birth weight in newborns [32]. Hematological effects include the induction of Fanconi anemia and leukemia [3,6,12]. Additionally, FA is also associated with cytotoxicity, genotoxicity [8,30], and mutagenesis [30,32].

Antioxidants reduce oxidative stress through various mechanisms, such as interacting with neutralizing free radicals, reducing the activity or the expression of free radical-producing enzymes, or increasing the activity or the expression of antioxidant enzymes [33]. Antioxidants can be natural, such as plant extracts, or synthetic [34,35]. They act by reacting with membrane phospholipids and inhibiting malondialdehyde (MDA), a product of lipid peroxidation [3,8,28,30,31,32], and by stimulating the production of endogenous antioxidant enzymes, such as catalase (CAT), which converts hydrogen peroxide into oxygen [5,7], and superoxide dismutase (SOD) [3,4,7,28,30,31,32], which neutralizes superoxide radicals [5,28], thus maintaining oxidant and antioxidant balance [4].

In this context, the aim of this research was to review the experimental data on the efficacy of antioxidant therapy in ameliorating the negative health effects associated with exposure to FA. Specifically, the study aimed to assess the efficacy of antioxidant interventions in reducing oxidative stress, protecting respiratory health, preserving neurological, renal, cardiac, and testicular function, and in facilitating detoxification mechanisms in FA-exposed individuals.

## 2. Materials and Methods

This systematic review was registered in the International Platform of Registered Systematic Review and Meta-analysis Protocols (INPLASY), protocol number 5825, with registration number INPLASY 202420005, and DOI number 10.37766/inplasy2024.2.0005.

We used the PICO format for the search, where P is FA-induced oxidative stress, I is antioxidants, C is the comparison with control, and O is the animal outcome.

The literature search was conducted in four databases: PubMed, Science Direct, SpringerLink, and Wiley, for articles published up to 1 September 2023, using a search strategy according to the Preferred Reporting Items for Systematic Reviews and Meta-Analyses (PRISMA) [36].

The literature review was performed independently by two groups of authors (L.B.U., R.Ș.S., C.M.G., and C.A.), using the keywords ‘oxidative stress AND antioxidants’, ‘FA’, and ‘histology OR histopathology’ to identify and select full-text research articles. In addition, any disagreements regarding the selected articles were resolved through discussion, with a third group of authors (C.U. and C.G.P.) acting as arbitrators in cases where consensus could not be reached. In order to include all the available papers, additional search engines, such as Google Scholar, and references from the selected articles were used. The search procedure is described in Table 1. 

### 2.1. Study Selection

Due to the paucity of human studies, only animal studies were reviewed. Research articles (full text) on the benefits of antioxidants in reversing the effects of FA-induced oxidative stress in experimental models, with biochemical and histological descriptions, were also included. The English-language articles that met these criteria, without any time limitation, were included in the present study. The inclusion and exclusion criteria are listed in Table 2.

Outcomes were represented by the antioxidant effect of different substances on the tissues affected by FA exposure, FA and antioxidant doses, routes of administration, and the type of animal model. Studies that did not meet the above characteristics were excluded from full-text evaluation.

### 2.2. Data Analysis

Data were abstracted with respect to the following characteristics of the included studies: author, year, animal model, dose and route of administration, type of antioxidant, FA effect, and antioxidant effect. A meta-analysis was not performed because of the great heterogeneity of the studies with different experimental protocols, different formulations administered, and different outcomes of the experimental animals. The differences in study populations, interventions, outcomes, or settings may be so great that combining the results would not be meaningful.

### 2.3. Study Quality Assessment

The SYRCLE risk of bias tool for animal research was used to assess the quality of each included study. The risk of bias was evaluated for each study, considering selection bias, performance bias, detection bias, attrition bias, and reporting bias.

## 3. Results

### 3.1. Article Selection

**Figure 1:**
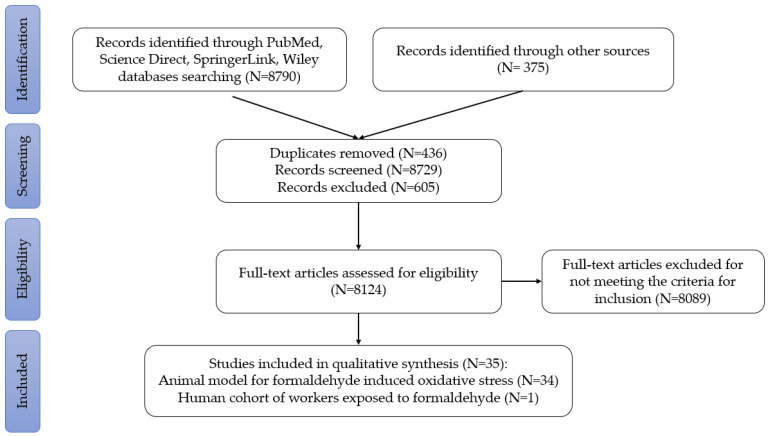
PRISMA flowchart for article selection.

There was a total of 9165 articles with the following distribution: 59 results on PubMed, 4691 from Science Direct, 1328 from SpringerLink, 2712 from Wiley, and 375 from article references. A total of 35 studies were selected for analysis. All were case–control studies with rats or mice randomly assigned to each study group, following a protocol approved by an ethical committee. Their distribution is shown in the PRISMA flowchart (Figure 1). 

### 3.2. Study Selection Bias

**Figure 2:**
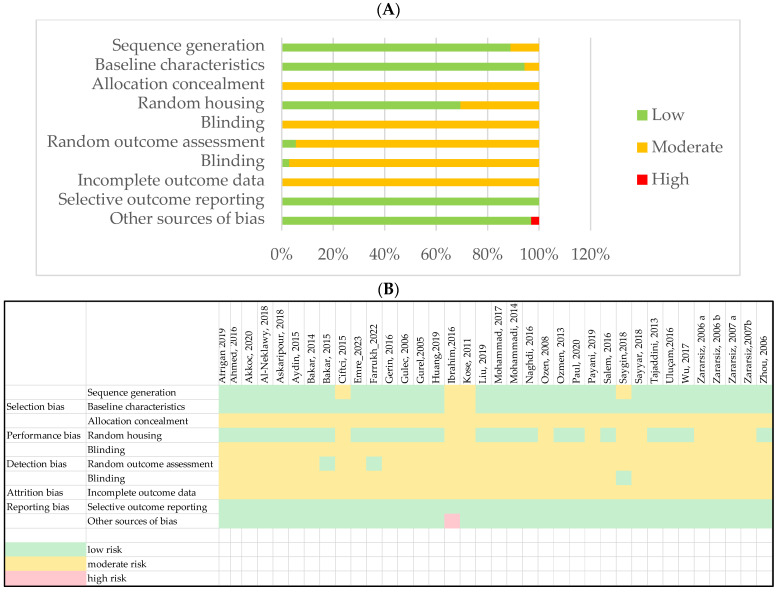
Assessment of risk of bias: (**A**) risk of bias graph; (**B**) risk of bias summary.

The most common bias was that the data on the blinding of animal allocation, the outcome assessment, and how the authors dealt with incomplete outcome data were not clearly reported in all the animal studies (Figure 2). FA was administered either intraperitoneally or by inhalation. 

Only one study did not describe the allocation of rats to different groups by randomization [1].

### 3.3. Antioxidants Effects 

Biochemical detection of MDA (malondialdehyde), CAT (catalase), SOD (superoxide dismutase), GPx (glutathione peroxidase), GSH (reduced glutathione), TOS (total oxidative state), TAS (total antioxidative status), NO (nitric oxide), MPO (myeloperoxidase), TSA (total sialic acid), and XO (xanthine oxidase) either serologically or from tissue homogenate were used to assess oxidative stress.

Apoptosis was detected by a TUNEL test or immunohistochemistry for Bcl-2 (B-cell lymphoma 2), Bax (Bcl-2 associated X protein), and caspase-3. The inflammatory process was assessed by serological cytokine levels (IL-1β, IL-6, IL-8, IL-10, TNF-α, or IFN-γ), histology, and immunohistochemistry.

#### 3.3.1. Antioxidants Pre-Treatment: Histopathological Effects of Antioxidants before FA Exposure

The present study has found that pre-treatment with pumpkin seed oil (PSO) or vitamin E may prevent liver, brain, and kidney damage, with a better outcome in the case of PSO administration [37] (Table 3). Pretreatment with lycopene reduced apoptosis intensity [28]. Pre-administration of *Matricaria chamomilla* has reduced apoptosis [34], while pre-administration of vitamin C in pregnant rats has prevented lung inflammation in the offspring [1].

#### 3.3.2. Effects of Concomitant Administration of Antioxidants during FA Exposure

Apoptosis intensity in the liver, heart, lungs, and kidneys is lowered by carnosine [4,7], melatonin [38], vitamin E [3,39], and *Matricaria chamomilla* [35].

FA-induced inflammation in the liver and lungs is decreased by co-administration of vitamin E [5], vitamin C [1], or ferulic acid [40]. 

Vitamin E, PA [41], or ferulic acid [40] administered concurrently with FA stop hepatocyte deterioration. Vitamin E [42,43], selenium [44], omega-3 fatty acids [30], or L-carnitine [45] when taken with FA appear to cause less neuronal damage. When *Matricaria chamomilla* [35], *Rosa damascena* [46], *Ficus carica* [47], proanthocyanidin [48], or vitamin E [49] are given, the testes appear to be protected from injury and their function appears to be improved. Eye damage caused by FA is prevented by co-administration of either *Nigella sativa* oil [2] or spirulina [50]. When FA was administered in combination with *Sarcococca saligna*, rats with rheumatoid arthritis showed less inflammation, pannus, and synovial hyperplasia [51]. Melatonin [31], vitamin E, or proanthocyanidin [39] may protect renal tubules from FA damage. The effects of concomitant administration of antioxidants are shown in Table 4.

#### 3.3.3. Effects of Administration of Antioxidants Following FA Exposure

Post-administration of melatonin [32], manganese chloride [54], or rose oil [55] may increase testicular function affected by FA administration, while post-administration of epigallocatechin-3-gallate (EGCG) [29] or Bronco-T [27] may reduce inflammation and apoptosis. The effects of post-administration of antioxidants are shown in Table 5.

#### 3.3.4. Effects of Administration of Antioxidants in Humans Following FA Exposure

In a study of 109 chemical plant workers exposed to formaldehyde for 1 to 25 years, FA toxicity was demonstrated and oral administration of Aevitum had anticlastogenic, antimutagenic, and anticarcinogenic effects [56].

#### 3.3.5. Mechanisms and Effects Induced by Antioxidants to Influence FA Exposure

Antioxidants are often used in therapy. Their effects include anti-inflammatory, anti-apoptotic, and antioxidant effects, as has been shown for curcumin [57,58,59,60,61,62], vitamin E [63], melatonin [64], lycopene [65], thymoquinone [66,67], ferulic acid [68,69], L-carnosine [70], and flavonoids [71]. Melatonin can repair DNA damage [64], while curcumin can maintain cell membrane integrity [60]. Lycopene regenerates vitamins C and E [65], thymoquinone requires nanoparticulate carriers [67], and ferulic acid protects DNA and lipids [69]. Various antioxidants directly scavenge ROS [59,62,63,64,65,67,68] or indirectly activate Nrf2 [61,64,68], and boost endogenous antioxidant defense mechanisms [60,62,63,64,65,66,67,68]. They also inhibit NF-kB [62,64,65,68] and reduce proinflammatory cytokines [60,61,62,63,64,65,66,67,68]. Curcumin induces apoptosis through the Bcl-2 pathway [60,62,63,65,66,68], while the effects of other antioxidants are not fully understood.

## 4. Discussion

This review summarizes the evidence from in vivo animal and human studies on the mechanisms and effects of antioxidants in combating FA exposure. The reviewed studies demonstrate the antioxidant, anti-inflammatory, and anti-apoptotic properties of various antioxidants. Notably, while antioxidants have been investigated for their potential to mitigate FA-induced damage, comprehensive reviews on their efficacy in treating FA-induced lesions are lacking.

Antioxidants are the primary defense against free radicals caused by oxidative stress. The literature reveals a paucity of human studies on the effects of antioxidants against FA, with only one notable study involving chemical plant workers currently available [56]. The difficulty in obtaining biopsies from organs other than nasal or oral mucosa in humans hampers clinical trials, making animal studies crucial to understanding the mechanisms and effects of antioxidant treatments.

### 4.1. Promising Antioxidant Treatments

The imbalance between oxidant and antioxidant systems triggers inflammatory responses and tissue damage. Antioxidants such as lycopene, *Matricaria chamomilla*, carnosine, melatonin, epigallocatechin-3-gallate, and proanthocyanidins have shown potential in protecting various organs from apoptosis [4,7,8,28,29,34,39]. In addition, antioxidants such as vitamin C, broncho-T, and ferulic acid have anti-inflammatory effects, although further studies are needed to identify other antioxidants with similar potential [1,27,40].

Several substances derived from organic foods and herbal remedies show potential as treatments for FA exposure due to their antioxidant, anti-inflammatory, and anti-apoptotic properties [35]. In addition to their antioxidant properties, some of them, such as *Matricaria chamomilla* [34,35], proanthocyanidins [39,41,48], ferulic acid [40], epigallocatechin-3-gallate from green tea [29], rose oil [55], pumpkin oil [37], and *Nigella sativa* [2], showed anti-inflammatory activity, while others such as proanthocyanidins [39,41,48], carvacrol [26], and ferulic acid [40] showed anticarcinogenic activity. Comparative studies suggest that some antioxidants, such as vitamin E, pumpkin seed oil, and lycopene, have significantly stronger effects than others, although further research is needed to validate these findings [37,39,65,72]. Lycopene has been described as a significantly more potent antioxidant than vitamin E (α-tocopherol), with approximately ten times higher antioxidant capacity [72], making it one of the most potent antioxidants available [65]. However, the precise classification and comparative efficacy of these substances require further investigation.

Omega-3 essential fatty acids are known for their antioxidant and neuroprotective properties [30]. Other antioxidants that may be beneficial in neurodegenerative diseases include lycopene from tomatoes [28], *Rosa damascena* extract [46], proanthocyanidins [48], curcumin [8], ferulic acid [40], epigallocatechin-3-gallate from green tea [29], selenium [54], melatonin [32], thymoquinone [52], and *Matricaria chamomilla* [34].

Melatonin is characterized by its ability to cross all body barriers, including the blood–testis barrier due to its hydrophilic nature [38,73,74]. Its lipophilic nature facilitates the penetration of cell membranes and organelles where it stimulates DNA repair enzymes, thereby preventing DNA damage [66,74]. The role of melatonin in reducing oxidative stress and apoptosis in infertility is noteworthy, as it is synthesized from serotonin in Leydig cells and affects androgen production and sperm formation [73,75,76]. It also inhibits apoptosis in testicular cells via the melatonin receptors MT1 and MT2 [75,77] and can protect the testes from damage post-treatment [32]. The present review has shown that even post-administration of melatonin can protect the testes from damage [32].

Vitamin E, present in Sertoli cells and spermatocytes, is essential for spermatogenesis and testosterone synthesis, which are impaired by oxidative stress [73]. Vitamin E supplementation has been shown to provide partial protection to the testes [49]. Similarly, vitamin C deficiency impairs spermatogenesis and testosterone synthesis, with deficiencies of both vitamins E and C leading to neurological damage [73,78]. The present review has shown that pre-treatment with vitamin E may protect the brain from cell loss [37]. Carvacrol, comparable to vitamins E and C, has significant antioxidant effects, crossing the blood–brain barrier and reducing nitric oxide levels, lipid peroxidation, and COX-2 activity [79].

Although antioxidant therapy does not restore normal tissue architecture after FA-induced damage, it serves as an effective adjunctive treatment [26,30,39,40,41,49,53]. As we found only three studies on the effect of antioxidants against FA exposure in humans, the present review focused on animal studies. This could be seen as a weakness of this work, which casts doubt on its overall validity in humans. However, the relevance of the present work lies in the analysis of a large number of tests, including both single-use and combinations of antioxidants, which showed some higher efficacy. The results of the present review support the idea that future administration of antioxidants in combination could greatly reduce the adverse effects of FA.

### 4.2. Bias and Limitations in Human Studies

Human studies of the effects of FA are subject to potential bias due to the lack of large cohort case–control studies and the limited number of human trials of antioxidants. Despite the large number of studies on FA-induced oxidative stress in humans [14,80,81,82,83,84,85,86,87,88,89,90,91,92,93,94,95,96], most have been cell culture studies [85,88,91,94] or literature reviews [14,80,82,83,87,89,90,91,92,93,95,96,97,98], with only a few original human studies [81,86,99] analyzing patients’ urine [81], blood [86,95], nasal lavage fluid [84], and blood and buccal mucosa cells [98]. Only one study evaluated histopathological changes in nasal mucosa biopsies [97]. The lack of comprehensive histopathological evaluation limits the clinical applicability of these findings.

### 4.3. FA Exposure

While endogenous FA is essential for functions such as DNA methylation and cellular metabolism, its excess can lead to genotoxicity, cytotoxicity, and various pathological conditions [11,24]. Intracellular pathways convert FA to less reactive molecules, maintain low levels of free formaldehyde, and recycle glutathione to prevent redox imbalance [12]. Elevated endogenous FA levels in cancer patients suggest an association with tumor progression, with FA oxidation enhancing ALDH activity [11,24,53].

On the other hand, FA is widely used in industry and medicine [5,32,42,44]. FA and its metabolites are rapidly transported to the lungs, kidneys, liver, hematogenous bone marrow, and brain [9,37,42], where they may react non-enzymatically with amino acids, proteins, DNA, RNA, and unsaturated fatty acids [8,32,37,44,49] or be enzymatically metabolized by the following enzymes: aldehyde dehydrogenase (ALDH), xanthine oxidase (XO), catalase (CAT), peroxidases, aldehyde oxidase (AO), glyceraldehyde-3-phosphate dehydrogenase (GAPDH), and specific NAD-dependent FA dehydrogenase [5,42,100]. FA can also increase oxygen activity in human tissues, leading to lipid peroxidation [9,39]. It can cause DNA damage [1,3,5] by generating ROS in mitochondria, leading to cell death [5] and inflammation [3].

According to Paul et al. (2020), the oral, intraperitoneal, or inhalation routes are mostly used in animal studies, considering that FA is rapidly distributed in several tissues such as the brain, testes, liver, and others [37].

Animal studies link FA exposure to brain-increased oxidative stress [8,30,101], an organ that is highly sensitive due to its high oxygen consumption and limited endogenous antioxidants [62,102,103]. The neurotoxicity of FA [30,104] has been linked to variable conditions, such as Parkinson’s and Alzheimer’s disease [8,28,43] and potential brain inflammation and cancers [105], e.g., astrocytoma, after 14 to 30 years of exposure [30,106]. While there are anecdotal reports of offspring malformations in rodents, the teratogenic effect remains unproven [83].

It was shown that rats exposed at >6 ppm and a reduced proportion of mice exposed at >14 ppm, for more than two years, developed nasal squamous cell carcinoma, preceded by squamous metaplasia. Gene alterations were observed only between 6 and 16 ppm, mainly involving pathways related to the cell cycle, DNA repair, and apoptosis. Squamous metaplasia, an adaptive response to cytotoxicity, provides some protection at FA concentrations above 6 ppm, but this is insufficient to prevent cytotoxicity [13]. However, despite p53 mutations in rats exposed to FA for more than two years, the direct link to carcinoma remains unclear [13]. Chronic exposure to FA at 10–15 ppm enhances cell proliferation and significantly increases nasal tumor incidence in inhalation bioassays [13]. The majority of the experiments in this review used 10 ppm FA, with no animals developing carcinoma within the two-week exposure period [4,7,26,55].

A carcinogenic effect of FA has also been reported in humans, but the results were not statistically significant [25]. A possible source of error could be its partial elimination by conversion to carbon dioxide [14,83], resulting in a reduced FA tissue concentration of less than 7% [89]. Genotoxicity cannot be excluded as an effect of FA toxicity [107], while trisomy or tetrasomy of chromosomes 4, 5, 15, and 17 were found in exposed workers in a study [108].

Exposure to FA poses significant health risks, including corrosive lesions, irritation, hypersensitivity, carcinogenicity, and reproductive risks. Higher concentrations are found in laboratory preparation and storage rooms, with teachers being more exposed than students [109,110]. Symptoms include eye and respiratory tract irritation, fatigue, headache and dizziness, and inability to concentrate [86,110], with rare cases of wheezing but normal IgE levels [25,86]. Despite the irritating nature of FA, human studies have not shown a significant increase in the risk of nasal cancer [83].

Corrosive lesions of the stomach and esophagus and rare lesions of renal insufficiency have been reported with substantial FA ingestion [25]. Due to its high irritant effect on the eyes, nose, and skin, FA could not be administered to humans at doses higher than 5 ppm [25]. Although hypersensitivity and asthma are very rare in humans, FA has been shown to induce type IV hypersensitivity [25]. Its inhalation is irritating, especially to throat tissues [25].

Female exposure to FA may be associated with menstrual disorders and endometriosis, while exposure during pregnancy may result in prematurity and low birth weight or birth defects [92].

**Figure 3:**
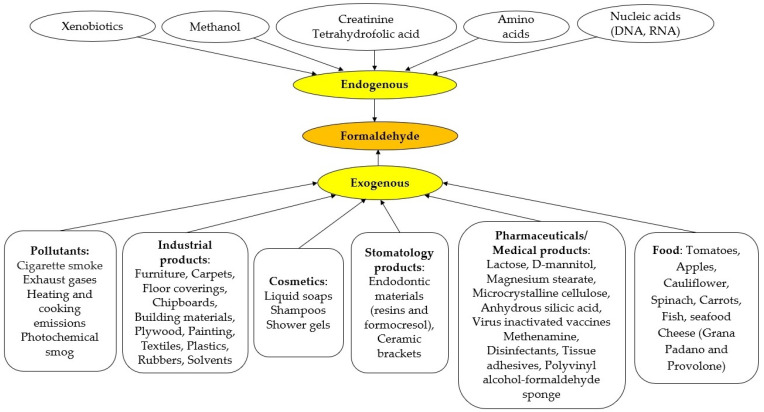
Sources of formaldehyde (FA) (after [1,2,3,4,8,10,11,12,21,22,23,24,28,30,31,32,52,111,112,113,114,115,116,117,118,119,120,121,122,123,124,125,126,127].

The accepted occupational exposure limit is 0.5 mg/m^3^ [3], while the indoor exposure limit is 0.08 mg/m^3^ [43], with a maximum accepted exposure of 100 mg/kg [37]. Sources of exposure to FA are shown in Figure 3.

### 4.4. Metabolism and Endogenous FA Regulation

Formaldehyde (FA) is naturally present in almost all human cells [128] and plays a crucial role in physiological processes but poses a risk if it accumulates in excess. It is metabolized to formic acid by enzymes such as formaldehyde dehydrogenase (FDH), which requires glutathione as a cofactor [22,23]. Rapid metabolism and excretion prevent significant accumulation even at relatively high exposure levels [23]. Maintaining a balance between FA production and degradation is essential for cellular homeostasis. Disruption of this balance can lead to FA accumulation, resulting in cytotoxic effects, oxidative stress, DNA damage, and various pathological conditions [11,22,24].

Approximately 40% of FA is in its free form [22]. Plasma formaldehyde levels are typically 2.5 ppm [23]. The odor of FA is detectable at low concentrations (up to 1 ppm) and becomes irritating at concentrations above 2 ppm [23]. It has a short half-life of about 1.5 min [22,23]. High levels of FA can deplete glutathione, thereby increasing the toxicity of FA, particularly through dermal, gastrointestinal, or respiratory exposure [22]. Despite rapid metabolism and excretion, FA does not accumulate significantly in plasma, even when inhaled at high doses (14 ppm) [23]. However, exposure to 5 ppm may cause changes in the liver and lungs, while 10 ppm may affect the brain, liver, lungs, kidneys, and testes [11,22,23].

At higher concentrations, FA can cause cytotoxicity, necrosis, and carcinogenic effects by interacting with proteins, nucleic acids, and unsaturated fatty acids, leading to inflammatory and allergic reactions, protein denaturation, and increased free radical production, thereby accelerating apoptosis or necrosis [24]. Elevated endogenous FA levels in cancer patients suggest that tumor tissues release this compound, possibly due to increased biosynthesis and altered FA clearance mechanisms in tumor cells, which may enhance ALDH activity [129]. Reducing FA synthesis or increasing its metabolism may delay cancer progression [130].

Intracellular FA levels are tightly controlled by metabolic pathways that convert FA to less reactive molecules such as formate. The cytosolic metabolism of FA begins with its reaction with glutathione (GSH) to form S-hydroxymethyl-GSH (HSMGSH), which is then oxidized by alcohol dehydrogenase 5 (ADH5) to S-formylglutathione. This compound generates formate by hydrolysis, maintaining low intracellular FA concentrations and recycling GSH to prevent redox imbalance. Malondialdehyde (MDA), released during the oxidative breakdown of polyunsaturated lipids, serves as a biomarker of oxidative stress and is cytotoxic due to its aldehyde groups [24]. The reduction in GPx levels may be due to its use in reactions catalyzed by FDH [29]. Cytochrome P450 recognizes FA as a substrate, potentially activating enzymes that generate reactive oxygen species (ROS), leading to the damage of membranes, proteins, and nucleic acids [34,44]. In addition, FA exposure reduces the activity of antioxidant systems such as CAT, SOD, and GSH, leading to oxidative stress [5,37]. Consequently, as endogenous antioxidant systems struggle to counteract the negative effects of FA, exogenous antioxidants may be required as adjuvant therapy.

Formaldehyde is generated through mitochondrial serine hydroxy-methyl-transferase and serine oxidation, with another significant source being creatine metabolism, where semi-carbazide-sensitive amine oxidases (SSAOs) produce FA, hydrogen peroxide, and ammonia. SSAOs are abundant in vascular endothelial and smooth muscle cells, with serum levels rising in pathological conditions [11].

FA also contributes to DNA methylation, which is critical for cellular memory. While certain levels of FA promote cell proliferation and memory formation, excessive synthesis or metabolic abnormalities can lead to age-related memory loss and neuronal damage [30,130]. Since exogenous FA does not accumulate in the body and endogenous FA becomes dangerous at critical levels, antioxidant treatments could potentially mitigate its harmful effects. However, the precise limit at which endogenous FA becomes dangerous has not yet been established.

In conclusion, while endogenous FA is essential for normal physiological functions, excessive levels due to metabolic imbalances or increased synthesis pose significant health risks, contributing to diseases such as cancer, atherosclerosis, and age-related cognitive decline. Further research into FA metabolism and its effects on health is essential to develop effective mitigation strategies.

## 5. Limitations and Future Directions

This review has several limitations, including the short duration of formaldehyde (FA) and antioxidant administration in animal models, a lack of comparative studies on antioxidant effects across different organs and antioxidants, and insufficient human studies on antioxidant administration before, during, or after FA exposure.

Although it has been shown that the rhesus monkey model for nasopharyngeal carcinogenesis is similar to the rodent model for FA exposure, extrapolating these results to humans is challenging due to ethical constraints on exposing humans to high concentrations of FA [13,15]. Additionally, there are significant anatomical and physiological differences between rodents and humans [13]. For instance, while rats breathe exclusively through their noses, humans can breathe through both their noses and mouths, potentially leading to different areas of the body being affected by FA inhalation [13]. Moreover, humans have weaker endogenous formaldehyde scavenging abilities compared to rodents [24].

Despite these limitations, this review underscores the potential of antioxidant treatments to mitigate FA-induced damage and emphasizes the need for further research to determine optimal delivery strategies and dosages.

## 6. Conclusions

In conclusion, while animal studies provide valuable insights into the mechanisms and effects of antioxidants against FA exposure, human studies are limited. Antioxidants offer promising protective effects against FA-induced oxidative stress, inflammation, and apoptosis. Further research is essential to elucidate the precise mechanisms, comparative efficacy, and optimal use of antioxidants in mitigating the adverse health effects of FA exposure.

## Figures and Tables

**Table 1:** Search terms.

Theme	Keywords and Boolean Descriptors
Oxidative Stress	“Oxidative Stress” AND “Antioxidants”
Formaldehyde	“Formaldehyde”
Histology	“Histology” OR “Histopathology”

**Table 2:** Inclusion and exclusion criteria.

Criteria	Inclusion	Exclusion
Type of reference	Full-text articles, research articles	No full text, books/chapters, conference articles, review
Language	English language	Non-English language
Type of study	In vivo studies	In vitro studies
Outcome	Studies focused on antioxidant effects	Studies not related to antioxidant effect

**Table 3:** The effects of antioxidants pre-treated in FA exposure.

Organ	Animal Model/ References	Antioxidant and Administration in Relation to FA	Outcome in Comparison with FA Administration by Biochemical and Histopathological Analysis
Liver	Mouse [37]	pumpkin seed oil 4 mL/kg p.o. + FA 10 mg/kg i.p. vit E 4 IU/kg p.o. + 10 mg/kg FA i.p., daily, 28 days	- normal liver architecture - less intense changes in vitamin E group than in pumpkin seed oil group - ↓ MDA, ↓ GOT, ↓ GPT
Brain	Rat [28]	lycopene 10 mg/kg p.o., daily, 12 weeks + FA (10 or 20 ppm) inhalation, 6 h, 5 days/week	- ↓ apoptosis: ↓ survivin, Bcl-2 - ↓ NO, ↓ MDA, ↓ CAT - ↑ neurotransmitters
	Mouse [37]	pumpkin seed oil (4 mL/kg) p.o. + FA 10 mg/kg i.p. vit E 4 IU/kg p.o. + 10 mg/kg FA i.p., daily, 28 days	- ↑ astrocyte number - hippocampus recovery - less intense changes in vitamin E group than in pumpkin seed oil group - ↓ MDA
	Rat [34]	*Matricaria chamomilla* (200 mg/kg, 500 mg/kg) 1 h after FA 10 mg/kg i.p., 30 days	- ↓ apoptosis in hippocampus - ↓ MDA, ↑ TAC
Lung	Pregnant rat and offspring [1]	- pregnant rats—vitamin C (150 mg/kg) by gavage for 1 h before each FA exposure (0.92 mg/m^3^, 1 h/day, 5 days/week) during 21 days of gestation - offspring—i.p. injection of lipopolysaccharide (LPS, *Salmonella abortus equi*, 5 mg/kg, i.p.)	- ↓ MPO, - ↑IL-6, ↓ IL-10, ↓ IFNϒ
Kidney	Mouse [37]	pumpkin seed oil p.o. + FA i.p. vit. E p.o. + FA i.p., daily, 28 days	- normal kidney architecture - less intense changes in vitamin E group than in PSO group - ↓ MDA, ↓creatinine

↑: increased; ↓: decreased; Bcl-2: Bcl-2 (B-cell lymphoma 2); CAT: catalase; i.p.: intraperitoneally; FA: formaldehyde; GOT: glutamic oxaloacetic transaminase; GPT: glutamic pyruvic transaminase; MDA: malondialdehyde; NO: nitric oxide; p.o.: per os; ppm = part per million; vit.: vitamin.

**Table 4:** The effects of antioxidant concomitant administration in FA exposure.

Organ	Animal Model/ Reference	Antioxidant and Administration in Relation to FA	Outcome in Comparison with FA Administration by Biochemical and Histopathological Analysis
Liver	Rat [26]	carvacrol 20 or 40 mg/kg i.p. once every 48 h + FA inhalation (10 ppm/8 h) 5 days/week, daily 4 weeks	- ↓ TOS
	Rat [40]	ferulic acid 50 mg/kg i.p. + FA 10 mg/kg i.p., daily, 10 days	- ↓ IL-6, ↓ TNF-α, ↓ IL-1β, ↓ IL-8, - degeneration - ↑ CAT, ↑ SOD, ↑ GPx - ↓ MDA
	Rat [41]	proanthocyanidin 100 mg/kg i.g. + 10 mg/kg FA i.p. vitamin E 30 mg/kg i.g. + 10 mg/kg FA i.p., daily, 14 days	- hepatocyte hypertrophy, ↓ hepatocyte - degeneration, normal hepatic structure - ↑ SOD, ↑ GPx, ↓ MDA, ↓ TSA
	Rat [5]	vitamin E 300 mg/kg i.m. + FA 10 mg/kg i.p., daily, 10 days	- ↓ XO, ↓ NO - prevented protein oxidation
	Rat [7]	carnosine 100 mg/kg p.o. daily + FA 5.27 ± 0.24 or 10.02 ± 0.16 or 15.2 ± 0.19 ppm inhalation, 5 days/week, 4 weeks	- ↓ apoptosis - ↑ TAS, ↓ TOS, ↓OSI
	Rat [4]	carnosine 150 mg/kg/day p.o. + FA 10 ppm inhalation 8 h/day, 5 days/week, 4 weeks	- ↓ apoptosis - ↑ TAS, ↓ TOS
Brain	Rat [52]	thymoquinone in corn oil 20 mg/kg i.g. + FA 10 mg/kg diluted in 10% i.p., daily, 15 days	- ↑ CAT, ↑ GPx, ↑ SOD - less subarachnoid hemorrhage, no - intracerebral hemorrhage
	Rat [42]	vitamin E 300 mg/kg i.m. + FA 10 mg/kg i.p., daily, 10 days	- less neuronal damage - ↓ MDA
	Mouse [43]	vitamin E 50 mg/kg i.g. + 0.155 mg/kg/day FA + 0.193 mg/kg/day PM2.5 intranasal instillation, daily, 7 days	- less neuronal damage - ↓ ROS, ↓ COX-2 - ↑ GSH, ↑ SOD
	Rat [8]	curcumin 100 mg/kg i.g.+ FA 9 mg/kg i.p., daily, 2 weeks	- ↓ MDA - ↑ TAC
	Rat [38]	melatonin 25 mg/kg i.p. + FA 10 mg/kg i.p., daily, 14 days	- ↓ Bax-stained cells - ↑ SOD, GPx, ↓ MDA
	Mouse [44]	selenium 0.1, 0.2, 0.4, 0.8 mg/kg i.p. + FA 10 mg/kg i.p., daily, 14 days	- less neuronal damage
	Rat [31]	omega-3 fatty acids 400 mg/ kg i.g. + FA 10 mg/ kg i.p., daily, 14 days	- reduced damage to neurons - ↑ SOD, ↑ GPx, ↓ MDA
	Rat [45]	L-carnitine 0.5, 1 g/kg i.p. + FA 10 mg/kg FA diluted with 10% PBS i.p., daily, 14 days	- reduced neuronal degeneration - ↑ SOD, ↑ GSH, ↓ MDA
Heart	Pregnant mouse [3]	vitamin E 0.1 μg i.p. + FA 0.5, 1, 1.5 mg/kg FA 40% (*w*/*w*) in aqueous solution i.p., daily, 21 days	- ↓ apoptosis - ↑ SOD, GSH, ↓ MDA
Lung	Rats [7]	carnosine 100 mg/kg p.o. daily + FA 5.27 ± 0.24 or 10.02 ± 0.16 or 15.2 ± 0.19 ppm inhalation, 5 days/week, 4 weeks	- ↓ apoptosis - ↑ TAS, ↓ TOS
Testis	Rat [35]	*Matricaria chamomilla* 200 mg/kg or 500 mg/kg i.p. + FA 10 mg/kg i.p. daily, 30 days	- ↑ testosterone, sperm motility, and viability, depending on dose - ↓ apoptosis - ↓ MDA
	Mouse [46]	*Rosa damascena* 10, 20 or 40 mg/kg p.o. + FA 10% 10 mg/kg of i.p., daily, 40 days	- ↑ testosterone level, diameter of - seminiferous tubules, depending on dose - ↑ Leydig and germ cell number
	Mouse [47]	*Ficus carica* 200 mg/kg p.o. daily + 10 mg/kg FA (1/10) i.p. twice/day, 14 days	- quite normal spermatogenesis
	Rat [48]	proanthocyanidin 100 mg/kg i.g. + FA 1/10 diluted 10 mg/kg i.p., daily, 14 days	- IHC: ↑ testosterone in Leydig cells - ↓ MDA
	Rat [49]	vitamin E 30 mg/kg/day p.o. + FA 10 mg/m^3^ (12 h/day) by inhalation daily, 2 weeks	- partially prevent testicular damage - ↑ SOD, ↑ GPx, ↑ GSH - ↓ MDA
Cornea	Rat [2]	*Nigella sativa* oil 40 mg/kg i.g. + FA 10% 2 h/day inhalation, 5 days/week, for 2 weeks	- normal appearance
	Rat [50]	spirulina dissolved in distilled water p.o. of 400 mg/kg daily + 10% FA inhalation for 2 h/day 5 days per week, 2 weeks	- cornea similar to control group
Joints	Rat with rheumatoid arthritis [51]	*Sarcococca saligna* 250, 500, 1000 mg/kg p.o. + FA 0.1 mL 2% subplantar daily, 28 days	- ↓ synovial hyperplasia, ↓ pannus, - ↓ inflammation, depending on dose
Kidney	Rat [39]	proanthocyanidin 100 mg/kg p.o. + FA 10 mg/kg 1:10 with NS i.p. vitamin E 30 mg/kg i.g. FA 10 mg/kg 1:10 with NS i.p., daily, 14 days	- ↓ damage to kidney, ↓ apoptosis: ↑ Bcl2, ↓ Bax - protective effects on tubules—increased for vitamin E than for PA - ↓ GPx
	Rat [31]	melatonin 25 mg/kg i.p. + FA 10 mg/kg i.p., daily, 14 days	- minimal dilatation of distal tubules - ↑ SOD, ↑ GPx - ↓ MDA
	Rat [53]	omega-3 fatty acids 400 mg/kg i.g. + FA 10 mg/kg i.p., daily, 14 days	- ↓ damage to kidney - ↑ SOD, ↑ GPx - ↓ MDA
	Rat [4]	carnosine 150 mg/kg/day p.o. + FA 10 ppm for 8 h/day, 5 days/week by inhalation, 4 weeks	- ↓ TRPM2 positivity - ↑ TAS, ↓ TOS

↑: increased; ↓: decreased; Bax: BCL2-associated X, apoptosis regulator; CAT: catalase; COX-2: cyclooxygenase-2 inhibitors; FA: formaldehyde; GPx: glutathione peroxidase; GSH: reduced glutathione; h: hour; i.g.: intragastric gavage; IL-1β: interleukin-1β; IL-6: interleukin-6; IL-8: interleukin-8; i.m: intramuscular; i.p.: intraperitoneally; kg: kilograms; MDA: malondialdehyde; mg: milligrams; MPO: myeloperoxidase; NO: nitric oxide; OSI: oxidative stress index; p.o.: per os; ppm: parts per million; ROS: reactive oxygen species; SOD: superoxide dismutase; TNF-α: tumor necrosis factor-alpha; TAC: total antioxidant capacity; TAS: total antioxidative status; TOS: total oxidative state; TSA: total sialic acid; XO: xanthine oxidase.

**Table 5:** The effects of subsequent antioxidant administration following FA exposure.

Organ	Animal Model/ Reference	Antioxidant and Administration in Relation to FA	Outcome in Comparison with FA Administration by Biochemical and Histopathological Analysis
Brain	Mouse [29]	Epigallocatechin-3-gallate 20, 100, 500 mg/kg p.o. 1 h after 3 mg/m^3^ FA inhalation for 8 h, daily, 14 days	- ↓TNFα, ↓IL-1β - ↓ apoptosis: ↓caspase 3 - ↓ iNOS
Lung	Rat [27]	Bronco-T p.o. after 1 h FA 40% vapor environment Salbutamol p.o. 1 h after FA 40% vapor environment	- ↓ inflammation - ↑ SOD, ↑ CAT, ↑ G-6-PDH
Testis	Rat [55]	Rose oil inhalation 1 mL/1 h after FA 10 ppm/1 h inhalation, 35 days	- ↑ testosterone, Leydig cells - ↓ number of Leydig cells with damaged nucleus
	Rat [32]	Melatonin 25 mg/kg i.p. 1 h after FA 10 mg/kg, i.p., daily, 1 month	- ↓ Bax in testicular cells - ↑ SOD, ↑ GPx, ↓ MDA
	Mouse [54]	Manganese chloride 5 mg kg/day i.p. a week after FA 10 mg/kg twice per day i.p., 2 weeks	- ↑ sperm mobility and viability - ↑ diameters of testicular seminiferous and epithelial tubules

↑: increased; ↓: decreased; Bronco-T: poly-herbal formulation; CAT: catalase; EGCG: epigallocatechin-3-gallate; FA: formaldehyde; G-6-PDH: glucose-6-phosphate-dehydrogenase; GPx: glutathione peroxidase; h: hour; IL-1β: interleukin-1β; iNOS: inducible nitric oxide synthase; i.p.: intraperitoneally; kg: kilograms; m^3^: cubic meter; MDA: malondialdehyde; p.o.: per os; ppm: parts per million; SOD: superoxide dismutase; TNF-α: tumor necrosis factor-alpha.
